# Spinal Anesthesia for Renal Transplantation in Lung Resected Patient: A Case Report

**Published:** 2018-03

**Authors:** Nilofar Massoudi, Farhad Safari, Kamran Mottaghi

**Affiliations:** 1 Clinical Research and Development Unit at Shahid Modarres Hospital, Department of Anesthesiology, Shahid Beheshti University of Medical Science, Tehran, Iran; 2 Department of Anesthesiology, Loghman Hospital, Shahid Beheshti University of Medical Sciences, Tehran, Iran

**Keywords:** Regional Anesthesia, Renal Transplantation, Single lung

## Abstract

Renal transplantation is among the definitive therapies for treatment of patients with “End-Stage Renal Disease” (ESRD). Proper anesthesia should be considered in patients who undergo renal transplantation. On the other hand, anesthesia in patients with single lung is an ever challenging issue. In this case report, we introduce a 42 year old woman with “Autosomal Dominant Polycystic Kidney Disease” (ADPKD) who was candidate for renal transplantation and underwent regional anesthesia since she had one lung. The patient had bilateral renal resection 4 years ago (due to ADPKD) and was undergoing dialysis 3 times weekly. Thirty five years ago, left lung resection had been done for the patient (due to suspected Tuberculosis). This patient demonstrated our experience in management of regional anesthesia as a safe method in patients with single lung who would undergo renal transplantation.

## INTRODUCTION

Autosomal dominant Polycystic Kidney Disease (ADPKD) presents with numerous kidney cysts which result in kidney enlargement and presents with pain, hypertension, renal dysfunction and finally kidney failure (1,2).

ADPKD is one of the causes of End-Stage Renal Failure (ESRF). In a recent study, the incidence of ADPKD is estimated to be 1:400 to 1:1000 (3). Renal transplantation is the most acceptable treatment option for patients with ESRD; although, it may impose additional risk of diseases such as tuberculosis (4,5). Proper anesthesia should be considered in patients who undergo renal transplantation. Hypoxemia at rest and at post-operative period along with pre-operative hypercapnia make general anesthesia hazardous for single lung cases; although inspiratory and expiratory forces increase after renal transplant (6). The anesthesia should achieve hemodynamic stability, enhance perfusion of the transplanted kidney, and counteract the increased concentration of circulating catecholamines (7–9). As spinal (T10-L1) and vagus nerves transfer renal painful stimuli, a combination of general and regional anesthesia is preferred to regional anesthesia alone in patients who are candidate for renal transplantation (10,11).

In this case report we report a woman with ADPKD who was candidate for renal transplantation and underwent regional anesthesia alone since she had only one lung.

## CASE SUMMARIES

**Figure 1.**
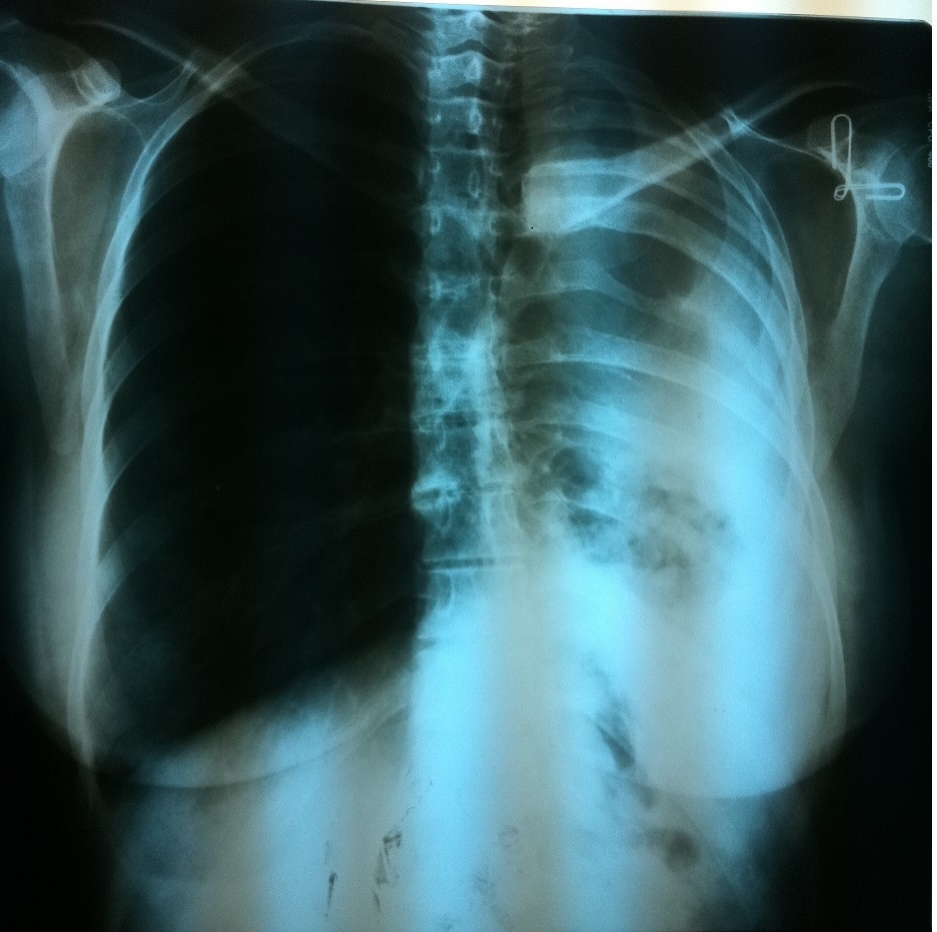
Patient's Chest X-Ray

The patient was a 42 year old woman (height 160 cm and weight 55 Kg), who had bilateral renal resection 4 years ago due to ADPKD and was undergoing dialysis 3 times weekly. Thirty five years ago, left lung resection had been done for the patient (due to suspected TB) (Figure 1). Drug history included daily folic acid, Erythropoetin alfa (Eprex) during dialysis and Calcium-Vitamin D every other day. Her brother had ADPKD as well who died after bowel resection. Electrocardiogram revealed sinus tachycardia, right axis deviation, “Left Posterior Hemiblock” (LPHB), Q wave in inferior leads. Echocardiography revealed ejection fraction of 55%, with trivial mitral valve prolapse and trivial mitral and tricuspid regurgitation. Respiratory consult revealed high risk for anesthesia and severe obstructive and restrictive lung disease. In spirometry, FEV1 (Forced Expiratory Volume in first second) =1.10 L/s, VC (Vital capacity) =1.68 L, and FEV1/FVC=81% were reported. Also ABG (Arterial Blood Gas) indices were as follow: pH=7.38, PaCO_2_=60 mmHg, PaO_2_=57 mmHg.

Laboratory findings were as follow: Na =135 mEq/L, K=4.6 mEq/L, Creatinine= 5.1 mg/dL, Hemoglobin = 13.3 g/dL, Platelet = 128000/μL^3^ (platelets per microliter), PT=15 seconds, PTT=27 seconds, INR=1.1, BT (Bleeding Time)=3 minutes, CT (Clotting Time)=6 minutes. No history about the coagulopathy or suspicious bleeding was reported. As the patient underwent dialysis the day before transplantation, potassium was 4.6 mEq/L.

The donor was a 30-year-old man who underwent laparoscopic nephrectomy under general anesthesia.

Using ultrasound guide and local anesthesia, Central Venous (CV) line was embedded through the right internal jugular vein. Before regional anesthesia, 300 mL normal saline infused via CV line and recipient underwent spinal anesthesia with 17.5 mg hyperbaric Bupivacaine 0.5% in L4-L5, lateral position and then the patient turned back to supine position. Anesthesia level was at T6 without any problem during surgery.

Before anesthesia the vital signs were as follow: Blood Pressure (BP)=110/70 mmHg, Heart Rate (HR) =108 beats per minute and CVP=8 cmH_2_O. Seven minutes after anesthesia, the HR became 64/min and BP=90/50 mmHg while the patient claimed that she had nausea. Then 0.6 mg intravenous atropine and 500cc normal saline were administered. After 3 minutes BP became 95/60 mmHg and HR=110/min.

During the surgery CVP was between 8 to 10 cmH_2_O. Before removing venous and arterial clamp, 1500 mL normal saline was administered for the patient and CVP=12 cmH_2_O and after declamping the renal vessels, urination was established for the patient. At the end of surgery, 50 μ fentanyl and 10 mg ketamine was administered to reduce potential postoperative pain. In the recovery room the vital signs were as follow: BP=110/60 mmHg, PR=110/min and urine output 500 mL.

## DISCUSSION

Renal transplantation is the choice method for ESRD cases. Previously, general anesthesia was the common anesthesia approach in these cases while nowadays regional anesthesia has been considered as preferred method as it avoids intubation and medications which are used in general anesthesia (12). In anesthesia management of patients who undergo renal transplantation, minimum toxicity should be considered. Length of surgery determines method of anesthesia. Due to sympathetic block in spinal anesthesia, preservation of blood pressure and intravascular volume is important. In this case, as we applied unilateral spinal anesthesia, severe bradycardia and hypotension did not occur.

In the study done by Kirdemir et al. due to unilateral spinal anesthesia, hemodynamic changes were low (13). One of the complications of spinal anesthesia up to T6 level is increased parasympathetic tone and irritability of bowels which could be controlled with atropine (12). In renal failure patient, administration of appropriate crystalloid is essential to correct their acidosis. We used saline to increase urine output.

The most important point is that our patient had one lung and anesthesia in these patients need special consideration.

FEV1 less than 2 liters is associated with post-operative respiratory complications so general anesthesia was not appropriate for our patient. On the other hand, fluid administration should be done precisely as these cases are prone to pulmonary edema (14–16). We did hydration by using the trend of changes in CVP to manage fluid administration.

Spinal anesthesia has some advantages: it is easy to perform and avoids tracheal intubation and decreases the risk of pulmonary complications and Deep Vein Thrombosis (DVT) (17). On the other hand, as blood pressure does not increase dramatically, blood loss and need for transfusion decreases by means of spinal anesthesia (17,18).

In a conclusion, regional anesthesia could be safe in cases with single lung who are candidate for renal transplantation.
